# Chemerin promotes angiogenesis in vivo

**DOI:** 10.14814/phy2.13962

**Published:** 2018-12-26

**Authors:** Nobuhisa Nakamura, Keiko Naruse, Yasuko Kobayashi, Megumi Miyabe, Tomokazu Saiki, Atsushi Enomoto, Masahide Takahashi, Tatsuaki Matsubara

**Affiliations:** ^1^ Department of Internal Medicine School of Dentistry Aichi Gakuin University Nagoya Japan; ^2^ Department of Pathology Nagoya University Graduate School of Medicine Nagoya Japan

**Keywords:** Akt PKB, angiogenesis, cell migration, chemokine, endothelial cell, extracellular signal‐regulated kinase (ERK), Chemokine‐like receptor 1(CMKLR1), C‐C chemokine receptor‐like 2(CCRL2)

## Abstract

Chemerin acts as a chemotactic factor for leukocyte populations expressing the G protein‐coupled receptor CMKLR1 (ChemR23). It is also an adipocytokine involved in obesity and metabolic syndromes. Previous studies have demonstrated that chemerin promotes angiogenesis in vitro, although the precise mechanism has not been elucidated. In this study, we have investigated whether chemerin regulates angiogenic processes and validated the associated mechanisms. In this study, chemerin stimulated angiogenesis in mice, which was demonstrated using Matrigel plug implantation assay, mouse corneal models of angiogenesis, and ex vivo rat aortic ring assay. To explore the mechanisms by which chemerin induced angiogenesis, we examined the effects of chemerin in human umbilical vein endothelium cells (HUVECs). Chemerin stimulated the differentiation of HUVECs into capillary‐like structures, promoted the proliferation of HUVECs, and functioned as a chemoattractant in migration assays. Chemerin induced the phosphorylation of Akt and p42/44 extracellular signal‐regulated kinase (ERK) in HUVECs and chemerin promotes angiogenesis via Akt and ERK. SiRNA against the chemerin receptor CMKLR1 but not that against another chemerin receptor, CCRL2, completely inhibited the chemerin‐induced migration and angiogenesis of HUVECs, which indicates that chemerin promotes the migration and angiogenic activities of HUVECs mainly through CMKLR1.

## Introduction

Chemerin, one of the chemoattractive proteins, is known as a retinoic acid receptor responder protein 2 (RARRES2) or tazarotene‐induced gene 2 protein (Tig2), whose expression is upregulated by the synthetic retinoid derivative tazarotene in primary cultures of keratinocytes and fibroblasts (Nagpal et al. 1997). Chemerin was also isolated as the natural ligand of the G protein‐coupled receptor (GPCR) CMKLR1 (also known as ChemR23) (Gantz et al. 1996; Wittamer et al. 2003; Bondue et al. 2011; Kulig et al. 2011). CMKLR1 was found to be highly expressed in plasmacytoid dendritic cells, macrophages, adipocytes, and endothelial cells. It also participates in attracting plasmacytoid dendritic cells and macrophages. Chemerin acts as a chemotactic factor for leukocyte populations expressing CMKLR1, such as immature dendritic cells (DCs), macrophages, and natural killer cells (Bondue et al. 2011). Another GPCR, C‐C chemokine receptor‐like 2 (CCRL2), has been identified as an additional receptor for chemerin by which chemerin enhances inflammation (Yoshimura and Oppenheim 2008).

Adipose tissues release a number of bioactive molecules that are generally called adipokines. Chemerin has also been identified as an adipokine involved in obesity and metabolic syndromes (Goralski et al. 2007). As an adipokine receptor, CMKLR1 has a role in adipogenesis and adipocyte maturation (Roh et al. 2007). Gene expression of chemerin was elevated in the adipose tissues of obese animals compared with lean animals and was markedly increased during differentiation of fibroblasts into mature adipocytes (Bozaoglu et al. 2010). Plasma chemerin levels are increased in patients and animals with obesity, coronary artery disease, and type 2 diabetes (Arita et al. 1999; Koenig et al. 2006; Qi et al. 2006; Parlee et al. 2012) and correlate with insulin resistance (Maeda et al. 1996; Yamauchi et al. 2001; Pajvani et al. 2003; Bozaoglu et al. 2007).

On the other hand, chemerin was found to be capable of stimulating angiogenesis in vitro (Bozaoglu et al. 2010; Kaur et al. 2010). It promoted capillary‐like structure formation by human umbilical vein endothelial cells (HUVECs) and functioned as a chemoattractant for HUVECs to promote migration and stimulated blood vessel growth (Bozaoglu et al. 2010; Kaur et al. 2010). However, the precise mechanisms and in vivo biological role of chemerin in the vasculature are still vague. In this study, to explore the effects of chemerin on endothelial cells, we have investigated whether chemerin stimulates migration, proliferation, angiogenesis in vitro, and angiogenesis in vivo.

## Materials and Methods

Animals – Male C57BL/6mice (WT) and Male Sprague–Dawley (SD) rats were obtained from Chubu Kagaku Shizai (Nagoya, Japan). All mice and rats were housed in individual cages under controlled temperature (24–1.0°C), on a 12‐h light/dark cycle and given standard laboratory mouse chow with water ad libitum. The Institutional Animal Care and Use Committees of Aichi Gakuin University approved all experimental protocols (AGUD 372).

Chemicals – Recombinant human chemerin (cat. No.: 2324‐CM), recombinant mouse chemerin (cat. No.: 2325‐CM), and VEGF‐A165 (cat.No:293‐VE) as positive controls were obtained from R&D systems (Abingdon, UK). Recombinant chemerin proteins are 137 aa mature segments that exert bioactivity.

Mouse Corneal Angiogenesis assay – Mouse corneal angiogenesis assay is a quantitative and reproducible assessment of angiogenesis in vivo. An advantage of this assay is that the measurement of background vessels is unnecessary because the vessels grow on an otherwise avascular tissue, and this also eliminates the possibility of vessel dilation being mistaken for angiogenesis (Rogers et al. 2007). Eight‐week‐old male C57BL mice were used for modified mouse corneal angiogenesis assay using previously described methods (Ouchi et al. 2004). A pocket, approximately 2–3 mm in size, was surgically prepared in the cornea extending toward a point 2 mm from the limbus. Poly‐HEMA (Sigma‐Aldrich; St Louis, MO) pellets containing chemerin (200 ng) or VEGF (100 ng, as a positive control), which enable slow release, were implanted into the corneal pockets on one side of the mouse (chemerin: *n* = 6, VEGF: *n* = 6). On day 7 after surgery, the mouse eyes were photographed, and cornea neovascularization was examined in a single‐blind manner. The angiogenic activity was evaluated based on the number of newly formed capillaries.

Mouse Matrigel Plug Assay – The formation of new vessels in vivo was evaluated using the Matrigel plug assay. Four hundred microliters of Matrigel (BD Biosciences; San Jose, CA) containing chemerin (10 nmol/L, *n* = 6), VEGF (5 nmol/L, *n* = 6), or vehicle was subcutaneously injected into the back of 8‐week‐old male C57BL mice. The mice were sacrificed 14 days after the injection. The Matrigel plugs with adjacent subcutaneous tissues were carefully recovered by en bloc resection and fixed in 4% paraformaldehyde. Immunohistostaining for CD31 (cat.No. sc‐52713) and von Willebrand factor (vWF, cat. No.: sc‐365712) (Santa Cruz Biotechnology; Santa Cruz, CA) were performed. The angiogenic activity was evaluated on the basis of the number of DAPI (+) CD31 (+) vWF (+) cells. Seven random microscopic fields per well were quantified. All assays were performed in triplicate.

Aortic ring assay – The descending thoracic aorta of single male 12‐week‐old Sprague–Dawley rat was isolated, cut into approximately 5‐mm segments, and embedded in Matrigel (BD Biosciences; San Jose, CA). The aortic rings were incubated with M199 plus chemerin (10 nmol/L, *n* = 6) or VEGF (5 nmol/L, *n* = 6). The culture medium was exchanged every 4 days. Two weeks later, vessel outgrowth was observed with phase‐contrast microscopy (Keyence Corporation, Osaka, Japan). Total tube length was calculated by the use of image analyzer software (Kurabo; Tokyo, Japan). The angiogenic activity was evaluated on the basis of the length of capillary per microscopic area. All assays were performed in triplicate.

Human Umbilical Vein Endothelial Cells – HUVECs were obtained from the American Type Culture Collection (ATCC, Manossas, VA). The cells were cultured in EGM‐2 MV complete medium (Lonza; Walkersville, MD).

Western Blot Analyses to Identify Chemerin Receptors in HUVECs – To identify chemerin receptors in HUVECs, confluent cultures of HUVECs were lysed with lysis buffer, subjected to SDS‐PAGE and detected using anti‐human CMKLR1 antibody (cat. No: sc‐374570) and anti‐human CCRL2 antibody (cat. No.: sc‐102423) (Santa Cruz Biotechnology; Santa Cruz, CA). For the detection of chemerin‐stimulated signaling in HUVECs, cells were stimulated with 10 nmol/L recombinant human chemerin for the indicated periods. A sample (20 *μ*g) of lysate protein was subjected to SDS‐PAGE and detected using anti‐phospho‐Akt (Ser473; cat. No.: #9271), anti‐Akt (cat. No.: #9272), anti‐phospho‐ERK (Thr202/Tyr204; cat. No.: #4376), and anti‐ERK (cat. No.: #4376, Cell Signaling Technology; Beverly, MA) antibodies.

Immunofluorescence staining of HUVECs – Cultured cells were fixed with 4% paraformaldehyde in 0.1 mol/L PBS, permeabilized with 0.05%‐Triton X‐100 in PBS, and stained with anti‐phospho‐Akt antibody, anti‐phospho‐ERK antibody (Cell Signaling Technology; Beverly, MA), and Alexa Fluor 568‐conjugated phalloidin (Invitrogen CO; Carlsbad, CA). Fluorescence was examined using a confocal fluorescence microscope (FV100, Olympus; Tokyo, Japan).

RNA Interference – The siRNA‐mediated knockdown of CMKLR1 and CCRL2 was performed according to previously described methods (Nakamura et al. 2009). Targeted nucleotides that effectively mediated the silencing of the expression of CMKLR1 (sequence: UAAAUUGCUAGUCCAAGGCtg) and CCRL2 (sequence: UUGUAAAGAGCCUUGCACGgt) were synthesized (Qiagen; Hilden, Germany). HUVECs were transfected with siRNAs or a 21‐nucleotide irrelevant RNA as a control using Hyperfect (Qiagen; Hilden, Germany) according to the manufacturer's protocol.

Boyden chamber assay – To assess the migration of HUVECs, we performed a modified Boyden chamber migration assay using previously described methods (Nakamura et al. 2009). The chambers were placed in 24‐well dishes filled with M199 containing 0.1% BSA with chemerin (0.1–10 nmol/L) or VEGF (5 nmol/L, as a positive control) in the lower chamber and incubated for 12 h. After incubation, the cells were labeled with CD31 and DAPI (Sigma‐Aldrich; St Louis, MO). The chemerin‐stimulated migratory capacity was then quantified by counting the migrated ECs on the lower surface of the filter using fluorescence microscopy (LAS AF, Leica; Leica, Germany). Seven random microscopic fields per each well were quantified. All assays were performed in triplicate.

Wound scrape assay – HUVECs were grown in 12‐well plates to a full confluent monolayer. In this condition, proliferation should be enough inhibited and therefore the wound healing should be due mainly to cell migration. The HUVEC monolayer was scraped in a straight line with a 200‐*μ*l pipette tip. Cell debris was removed by washing the cells once with 1 mL of growth medium, followed by incubation with 1 mL of medium supplemented with chemerin (10 nmol/L) or VEGF (5 nmol/L) as a positive control. A reference point was marked with a tip marker. The change rate of width between the leading edge covered by cells before and after 8 h of incubation was quantified. In addition to 24 h‐serum starvation, we have performed in a medium without serum for no more than 12 h.

Cell proliferation assay – When HUVECs reached 50% confluence in 24‐well dishes, cell growth was arrested using serum‐free EBM‐2 medium for 12 h. After preincubation with the phosphatidylinositol 3‐kinase (PI3‐K) inhibitor LY294002 (1 *μ*mol/L) or the mitogen‐activated protein kinase/ERK kinase (MEK) inhibitor PD98059 (5 *μ*mol/L), cells were incubated with chemerin (10 nmol/L) or VEGF (5 nmol/L); MTT (3‐(4,5‐dimethylthiazol‐2‐yl) ‐2,5‐diphenyltetrazolium bromide) (Sigma‐Aldrich; St Louis, MO) was added at a final concentration of 0.5 mg/mL, and after further 2 h of incubation, HUVECs were lysed with isopropanol containing 0.04 mol/L HCl. MTT reduction was read at 550 nm using a spectrophotometer (ARVO MX, Perkin Elmer Cetus; Norwalk, CT). For cell counting, HUVECs were detached from the plate and counted using a hemocytometer.

Tube formation assay in HUVECs – The tube formation assay was performed as previously described with the following modifications: 24‐well plates were coated with 1 mL of Matrigel and incubated at 37°C for 30 min to promote gelling. HUVECs were resuspended in growth medium (serum concentration 2%) and added to each well with chemerin (10 nmol/L) or VEGF (5 nmol/L: positive control) to a final volume of 2 mL. After 18 h, the plates were fixed, and morphology of the tubes was assessed. Total tube length per microscopic area was calculated using image analyzer software (Kurabo; Tokyo, Japan).

Statistical analysis – All the group values were expressed as the means ± SE. Statistical analyses were performed using one‐way analyses of variance (ANOVA). The level of significance was set at *P < *0.05 (SPSS ver.22.0, IBM; Armonk, NY).

## Results

**Figure 1:**
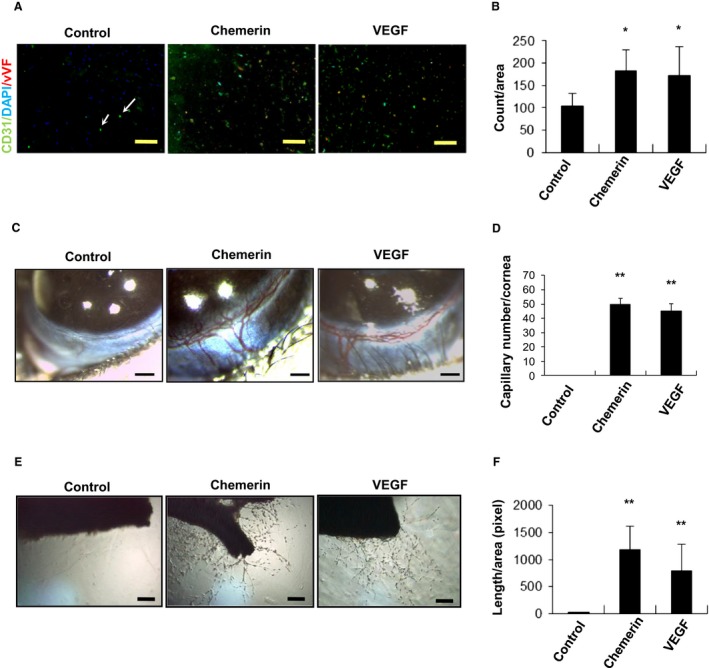
Chemerin promotes angiogenesis in vivo. (A) Matrigel plugs containing chemerin (10 nmol/L, *n* = 6), VEGF (5 nmol/L, *n* = 6), or phosphate‐buffered saline (control, *n* = 6) were injected subcutaneously into mice. Plugs were stained with the endothelial cell marker CD31 and vWF (arrow indicated). Bar = 100 *μ*m. (B) Endothelial cell infiltration of the plugs was assessed by immunohistochemical analysis of CD31 and vWF positive cells. The frequency of DAPI (+) CD31 (+) vWF (+) cells in five low power fields was determined for each Matrigel plug. The data were presented as the number of DAPI (+) CD31 (+) vWF (+) cells per microscopic area. The mean ± SD was derived from six Matrigel plugs for each condition. *P <* *0.05 compared with control. (C) Mouse corneal angiogenesis assay; Pellets containing chemerin (200 ng, *n* = 6), VEGF (100 ng, *n* = 6) were implanted in mouse cornea. Photographs of mouse eyes are shown (Control, chemerin 200 ng; VEGF, 100 ng). (D) The angiogenic activity was evaluated based on the number of newly formed capillaries per cornea. The results are shown as the mean ± SE **P <* *0.01 versus control. (D) Rat aortic ring assay; The descending thoracic aorta of single male Sprague–Dawley rat was removed, embedded in Matrigel, and incubated with M199 medium containing chemerin (10 nmol/L, *n* = 6), VEGF (5 nmol/L, *n* = 6). (E) Outgrowth of neovessels from the aorta was observed under phase‐contrast microscopy. (F) The length of neovessels from the aorta was calculated by the use of a cell image analyzer. **P <* *0.01 compared with control.

Chemerin Promotes Vessel Growth in vivo – To examine the effect of chemerin on angiogenesis in vivo, mouse Matrigel plug assay, mouse corneal angiogenesis assay, and rat aortic ring assay were performed. In the Matrigel plug assay, endothelial cell infiltration of the plugs was assessed by immunohistochemical analysis of CD31 and vWF positive cells (Fig. 1A). Quantitative analyses of histological sections revealed that the plugs containing chemerin displayed a significantly higher density (1.9‐fold) of CD31 and vWF positive cells (188 ± 45 counts/area) compared with that in the controls (102 ± 33 counts/area), which reached almost the same level as that seen with VEGF (*P *=* *0.043) (Fig. 1B).

The corneal assay is also an objective indication of angiogenic potential. In this study, neovascularization in corneal implants was markedly accelerated in the presence of chemerin (49 ± 4 capillary number/cornea) compared with that in the controls (no capillary) (*P *=* *0.001) (Fig. 1C). The stimulatory effect of chemerin was comparable with that of VEGF in this model (Fig. 1D). These data show that chemerin can promote neovascularization in vivo.

The outgrowth of neovessels from the descending thoracic aorta of male Sprague–Dawley rat was assessed using phase‐contrast microscopy (Fig. 1E). Quantitative analyses revealed that neovascularization was markedly accelerated in the presence of chemerin (1220 ± 950 pixel/area) compared with that in the controls (*P *=* *0.008) (Fig. 1F). The stimulatory effect of chemerin was comparable with that of VEGF. Ex vivo rat aortic ring assay also showed the angiogenic potential of chemerin.

**Figure 2:**
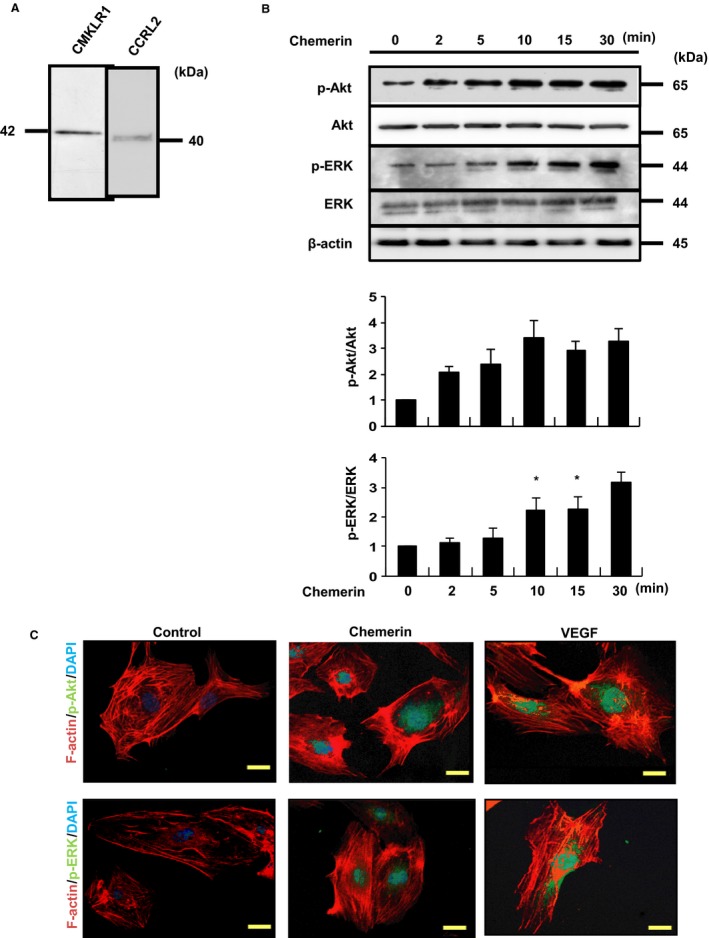
(A) Expression of chemerin receptors, CMKLR1 and CCRL2, in HUVECs. Chemerin receptors were identified by Western blot analyses using anti‐human CMKLR1 and CCRL2 antibodies. (B) The phosphorylation of Akt and ERK by chemerin. Chemerin stimulation (10 nmol/L) was conducted for the indicated time. The results of one of three experiments with similar results are shown. *P <* *0.01 versus control. (C) Confocal immunofluorescence analysis of HUVECs treated with chemerin or VEGF using rabbit mAb against phospho‐Akt (Ser473) and phospho‐ERK (Thr202/Tyr204) (green). Actin filaments have been labeled with Alexa Fluor 568‐conjugated phalloidin (red). Blue pseudo color = DAPI.

Identification of Chemerin Receptors – To examine whether vascular endothelial cells have chemerin receptors, we evaluated the expression of chemerin receptors including CMKLR1 and CCRL2 in HUVECs, using Western blot analyses. CMKLR1 was identified as a single band at 42 kD. We also detected a single band at 40 kD stained with anti‐human CCRL2 (Fig. 2A).

Chemerin Stimulates the Phosphorylation of Akt and ERK in HUVECs – In order to clarify the roles of Akt and MEK in angiogenesis, migration ability, and proliferative capacity, we have performed various experiments using inhibitors of PI‐3 kinase and MAPK/ERK kinase (MEK). As previously reported, chemerin significantly stimulated the phosphorylation of Akt and ERK in HUVECs in a time‐dependent manner (Fig. 2B). The phosphorylation of Akt and ERK was increased at 30 min after chemerin stimulation. Phospho‐Akt and phospho‐ERK were observed in and around the nuclei by immunofluorescence staining in HUVECs (Fig. 2C).

**Figure 3:**
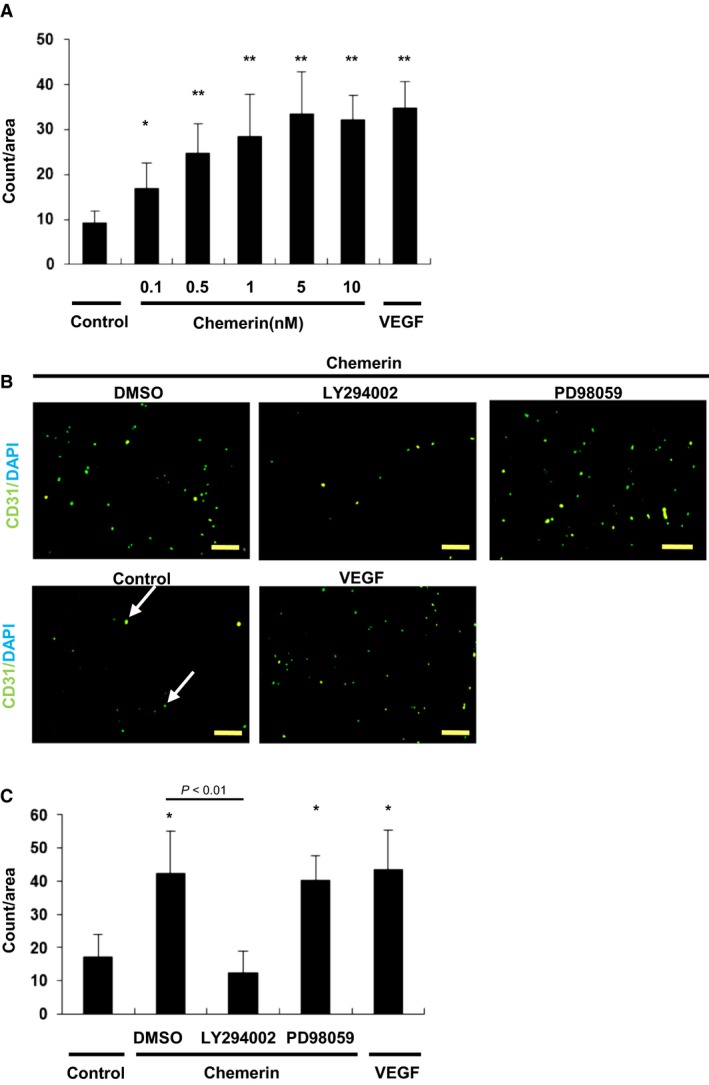
Migration of HUVECs induced by chemerin. (A) Modified Boyden chamber assay was performed with chemerin (10 nmol/L) or VEGF (5 nmol/L) as chemoattractant. Chemerin stimulation (10 nmol/L) was conducted for the indicated time. Seven random microscopic fields per each well were quantified. All assays were performed in triplicate. The results are shown as the mean ± SE. *P <* *0.05, **P <* *0.01 versus control. (B) Representative images of the migrated HUVEC in the modified Boyden assay. Cells were stained with CD31 and DAPI (arrow indicated). Bar = 100 *μ*m. (C) Chemerin‐induced enhancement of migration. After preincubation with LY294002 (1 *μ*mol/L) or PD98059 (5 *μ*mol/L), cells were incubated with chemerin (10 nmol/L). The migratory capacity was quantified by counting the migrated HUVECs on the lower surface of the filter. Seven random microscopic fields per each well were quantified. All assays were performed in triplicate. The results are shown as the mean ± SE. *P <* *0.01 versus control.

**Figure 4:**
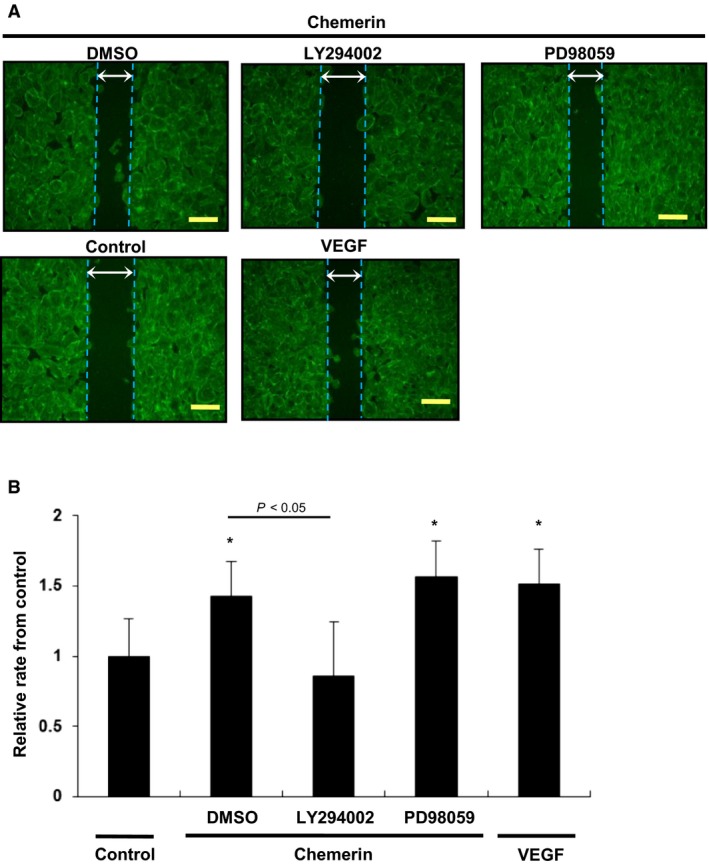
Migration of HUVECs stimulated by chemerin assessed by cell scratch assay. (A) Modified wound healing assay was performed with chemerin (10 nmol/L) or VEGF (5 nmol/L) as chemoattractant. Chemerin stimulation (10 nmol/L) was conducted for the indicated time. After preincubation with LY294002 (1 *μ*mol/L) or PD98059 (5 *μ*mol/L), cells were incubated with chemerin (10 nmol/L). (B) The change rate of width between the leading edge covered by cells before and after 8 h of incubation was quantified. Seven random wounds per each well were quantified. All assays were performed in triplicate. *P <* *0.05 versus serum‐free control.

Chemerin Promotes Migration of HUVECs via PI3‐K/Akt. – The number of migrated cells attracted by chemerin was 3.5‐fold higher (33.5 ± 10.4 counts/area at 5 nmol/L) than that under serum‐free conditions (9.7 ± 2.8 counts/area : *P *<* *0.007 vs. control). The migratory activities of HUVECs were increased by chemerin in a dose‐dependent manner (Fig. 3A). So, we determined the concentration of chemerin as 10 nmol/L for other experiments. Next, chemerin‐induced enhancement of migratory activities was completely inhibited by LY294002 (1 *μ*mol/L) (106% suppression: 42.7 ± 12.8 counts/area vs. 12.5 ± 6.4; *P *=* *0.023). On the other hand, the MEK inhibitor PD98059 (5 *μ*mol/L) did not inhibit the chemerin‐induced migration of HUVECs in modified Boyden chamber assays (42.7 ± 12.8 counts/area vs. 41.0 ± 7.6, *P *=* *0.026) (Fig. 3C). Similarly, LY294002 (1 *μ*mol/L) inhibited cell migration as assessed by an 8‐h cell scratch assay (1.42 ± 0.43 fold vs. 0.88 ± 0.85 fold from control: *P *=* *0.032); PD98059 (5 *μ*mol/L) did not inhibit the chemerin‐induced migration (1.42 ± 0.43 fold vs. 1.53 ± 0.72 fold from control) (Fig. 4A,B).

**Figure 5:**
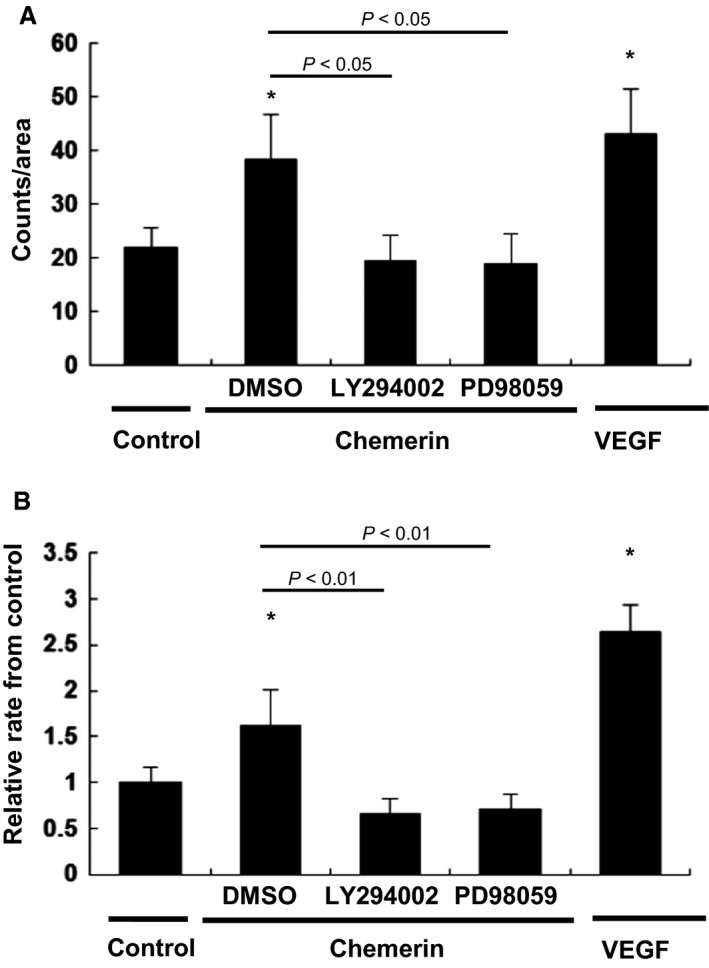
Chemerin promotes proliferation of HUVECs. Cell growth was arrested using serum‐free EBM‐2 medium for 12 h. After preincubation with LY294002 (1 *μ*mol/L) or PD98059 (5 *μ*mol/L), cells were incubated with chemerin (10 nmol/L) or VEGF (5 nmol/L). (A) Cell proliferation was assessed by counting cells with a hemocytometer. For cell counting, HUVECs were detached from the each seven plates and counted using a hemocytometer. All assays were performed in triplicate. (B) Cell proliferation was assessed by the MTT assay. MTT reduction was read by each 10 wells at 550 nm using a spectrophotometer. All assays were performed in triplicate. The results are shown as the mean ± SE. *P <* *0.05 versus control.

Chemerin Promotes Proliferation of HUVECs – Cell proliferation was assessed by counting cells with a hemocytometer and the MTT assay. Chemerin significantly increased the proliferation of HUVECs as shown by a 1.8‐fold increase in the number of cells (22.2 ± 4.3 counts/area vs. 39.3 ± 8.6 counts/area) (Fig. 5A). These effects were completely inhibited by LY294002 (1 *μ*mol/L) and PD98059 (5 *μ*mol/L) (39.3 ± 8.62 counts/area vs. 20.5 ± 4.8 counts/area (*P *=* *0.033), 39.3 ± 8.62 counts/area vs. 20.0 ± 5.4 (*P *=* *0.027); respectively). Similarly, chemerin increased the proliferation of HUVECs by 1.7‐fold increase in the MTT assay (1.73 ± 0.61 fold from control) (Fig. 5B). These effects were completely inhibited by LY294002 (1 *μ*mol/L) and PD98059 (5 *μ*mol/L) (1.73 ± 0.61 fold vs. 0.75 ± 0.33 fold (*P *=* *0.008), 1.73 ± 0.61 fold vs. 0.79 ± 0.32 fold from control (*P *=* *0.009); respectively), suggesting that chemerin stimulates cell proliferation via both PI3‐K/Akt and MEK/ERK.

**Figure 6:**
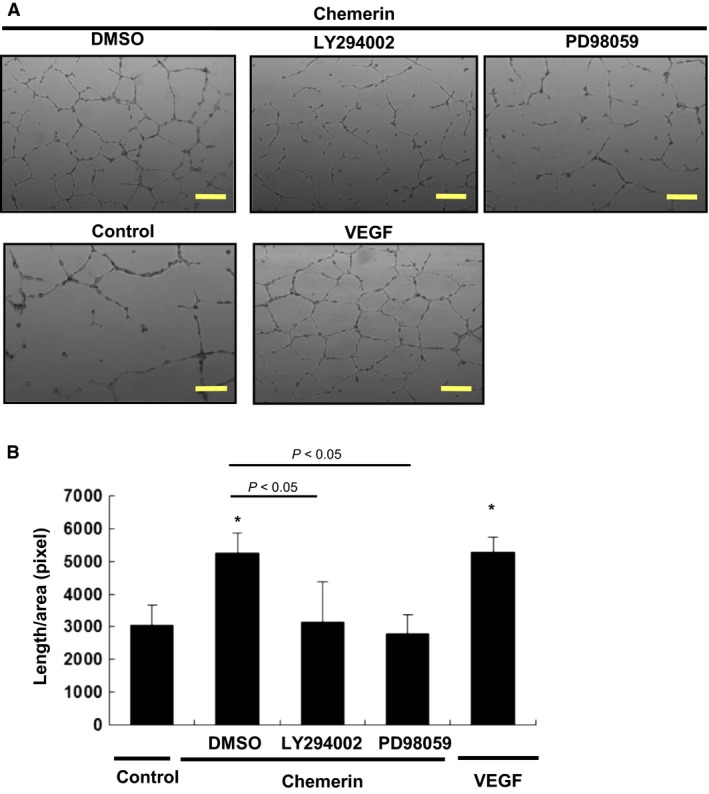
Chemerin promotes the differentiation of HUVECs into tube‐like structures. Tube formation assays were performed. HUVECs were seeded on Matrigel‐coated culture dishes in the presence of chemerin (10 nmol/L), VEGF (5 nmol/L), or BSA (10 g/mL: control). After preincubation with LY294002 (1 *μ*mol/L) or PD98059 (5 *μ*mol/L), cells were incubated with chemerin (10 nmol/L). (A) Representative cultures are shown. (B) Quantitative analysis of tube formation. Total tube length per each 10 microscopic area was calculated using image analyzer software. All assays were performed in triplicate. The results are shown as the mean ± SE. *P <* *0.05 versus control.

Chemerin Promotes Tube Formation of HUVECs – Chemerin induced the formation of tube‐like structures by HUVECs in Matrigel (Fig. 6A). This effect was markedly inhibited by LY294002 (1 *μ*mol/L) (5250 ± 750 pixel/area vs. 3220 ± 1340 pixel/area, *P *=* *0.042), and PD98059 (5 *μ*mol/L) (5250 ± 750 pixel/area vs. 2980 ± 450 pixel/area, *P *=* *0.031) (Fig. 6B), suggesting that chemerin stimulates angiogenesis via PI3‐K/Akt and MEK/ERK as well as cell proliferation.

**Figure 7:**
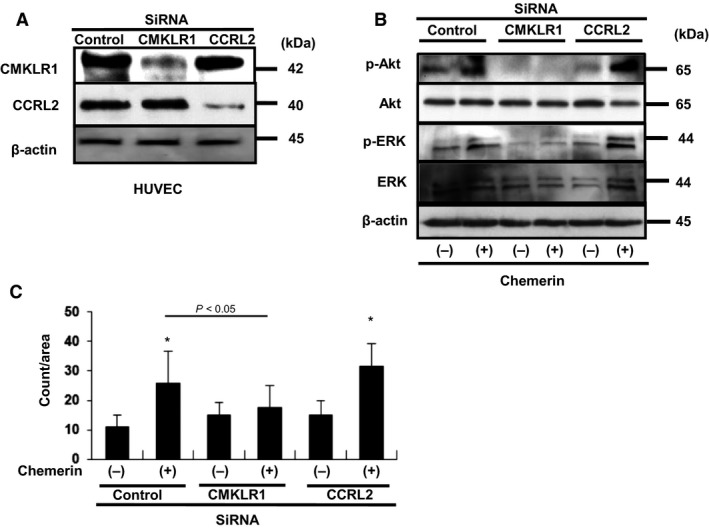
(A) The siRNA‐mediated knockdown of CMKLR1 and CCRL2. (B) Chemerin‐induced phosphorylation of Akt and ERK was inhibited by siRNA against CMKLR1. (C) Modified Boyden chamber assay was performed with chemerin (10 nmol/L) as chemoattractant. Chemerin‐induced enhancement of migration was inhibited by siRNA against CMKLR1. Seven random microscopic fields per each well were quantified. All assays were performed in triplicate. The results are shown as the mean ± SE. *P <* *0.05 versus control.

**Figure 8:**
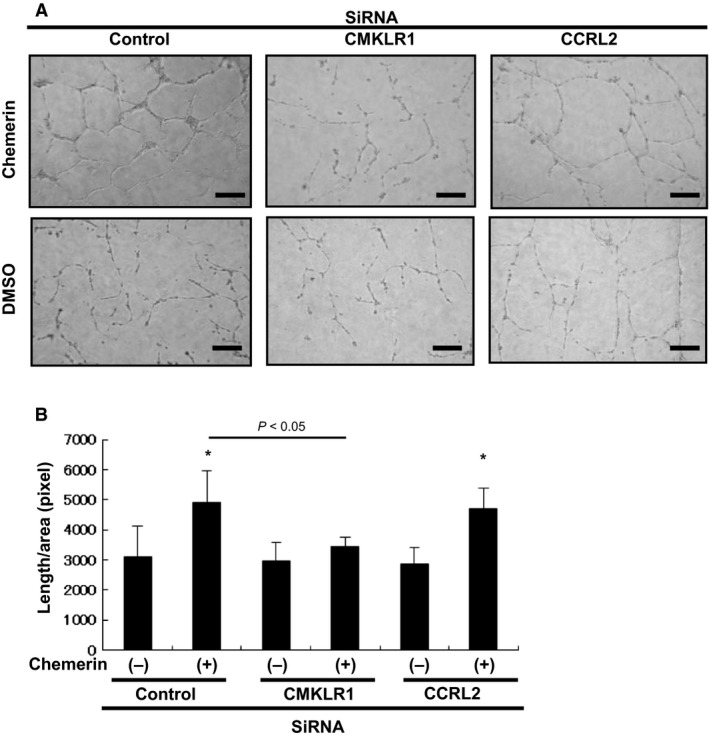
Chemerin promotes the differentiation of HUVECs into tube‐like structures via CMKLR1. HUVECs were seeded on Matrigel‐coated culture dishes in the presence of chemerin (10 nmol/L) Chemerin‐induced enhancement of angiogenesis was inhibited by siRNA against CMKLR1. (A) Representative cultures are shown. (B) Quantitative analysis of tube formation. Total tube length per each 10 microscopic area was calculated using image analyzer software. All assays were performed in triplicate. The results are shown as the mean ± SE. *P <* *0.05 versus control.

Knockdown of chemerin receptors – To elucidate the signaling pathways involved in chemerin‐stimulated migration and angiogenesis, targeted knockdown using siRNA of chemerin receptors was performed. Western blot analyses showed that transfection with siRNAs against CMKLR1 and CCRL2 effectively reduced the expression levels of the corresponding proteins by over 80% (Fig. 7A). As shown in Figure 7B, phosphorylation of Akt and ERK in HUVECs stimulated by chemerin was markedly inhibited only by siRNA against CMKLR1. Chemerin‐induced migratory activities were markedly inhibited by siRNA against CMKLR1 (26.9 ± 13.5 counts/area vs. 17.5 ± 7.7, *P *=* *0.044). However, siRNA against CCRL2 had no effect on chemerin‐stimulated migration (26.9 ± 13.5 counts/area vs. 32.5 ± 8.2). These observations indicate that chemerin stimulates migration via CMKLR1 (Fig. 7C). As shown in Figure 8, chemerin‐induced tube formation was completely inhibited by siRNA against CMKLR1 (4980 ± 1020 pixel/area vs. 3380 ± 230 pixel/area, *P *=* *0.021). However, siRNA against CCRL2 had no effect on chemerin‐stimulated tube formation (4980 ± 1020 pixel/area vs. 4780 ± 450; *P *=* *0.98) (Fig. 8B). These findings suggest that chemerin may play a role in migration and blood vessel formation of vascular endothelial cells via CMKLR1.

## Discussion

Chemerin shows positive correlation with various factors of metabolic syndromes (Zakareia 2012). Several reports have shown that the plasma chemerin levels are increased with obesity, coronary artery disease, and type 2 diabetes (Arita et al. 1999; Koenig et al. 2006; Qi et al. 2006; Parlee et al. 2012), suggesting that chemerin stimulates cardiovascular diseases (CVDs) and dysregulated angiogenesis. On the other hand, other reports have shown that chemerin stimulates angiogenesis in vitro (Rhee 2011) and that plasma levels of chemerin are positively correlated with the ankle/brachial blood pressure index (ABI) in diabetic peripheral vascular disease (Zakareia 2012). These suggest the potential angiogenic effect of chemerin. In this study, in vivo functional angiogenesis assays confirmed that chemerin significantly mediates the formation of blood vessels to a similar extent as that of VEGF. These results support the other reports, which show that the serum levels of chemerin and VEGF are significantly increased in the proliferative diabetic retinopathy and nonproliferative retinopathy patients (Du et al. 2016).

Chemerin is synthesized as a 163‐aa precursor (Storici et al. 1996; Nagpal et al. 1997; Wittamer et al. 2004; Zanetti 2004) and secreted as an 18‐kDa inactive proprotein to be cleaved into a 16‐kDa active form present in plasma and serum (Nagpal et al. 1997; Goralski et al. 2007). The 137 aa mature segment is known to bind to CMKLR1, resulting in the chemotaxis of macrophages and immature DCs (Luangsay et al. 2009). G protein‐coupled receptor 1 (GPR1) has been identified as a second receptor of chemerin. GPR1 acts as a decoy receptor for chemerin, and GPR1‐mediated activity has not been observed in primary cells or in vivo. GPR1 has not been reported to be expressed in leukocyte populations, but it is expressed in the central nervous system, skeletal muscle, skin, and adipose tissues (Barnea et al. 2008). Thus, we did not perform siRNA‐mediated silencing of GPR1 and did not investigate the GPR1‐mediated migration/angiogenesis.

CCRL2 was recently described as a third receptor for chemerin (Zabel et al. 2008). CCRL2 belongs to the chemokine receptor family. The expression of the human receptor has been reported in monocytes, macrophages, DCs, and other immune cells (Migeotte et al. 2002; Yoshimura and Oppenheim 2011), while the mouse receptor has been reported to be expressed only by DCs and macrophages (Otero et al. 2010). CCRL2 binds chemerin but does not support cell migration by itself (Monnier et al. 2012). Chemerin binds to human and mouse CCRL2 with high affinity similar to that for CMKLR1 and GPR1. However, the binding of chemerin to CCRL2 does not seem to mediate any signal transduction in cells and does not induce CCRL2 internalization. In this study, we provided evidence to support that the chemerin receptor CMKLR1 contributes to migration and angiogenesis induced by chemerin in ECs. We also firstly demonstrated that CCRL2‐siRNA did not affect the chemerin‐stimulated migration and angiogenesis in HUVECs. These data support the hypothesis that chemerin/CMKLR1 interaction also promotes angiogenesis in vivo (Monnier et al. 2012).

PI3‐K, which is a upstream of Akt, is one of the most important regulatory proteins involved in controlling several key functions of the cell, such as cell growth, aging, and transformation (Shaw et al. 1997; Sotsios and Ward 2000). Akt is an important regulator of various cellular processes including glucose metabolism and cell survival (Hattori et al. 2003; Xi et al. 2005). Chemerin is also reported to promote the formation of endothelial tubes in a MAPK‐dependent manner in fibroblast/endothelial cell cultures. (Zabel et al. 2006; Bozaoglu et al. 2007). The ability of chemerin to promote vascularization of endothelial cells can be abolished by an MEK inhibitor, which suggests that chemerin‐induced angiogenic effects are dependent on the MEK/ERK pathway (Bozaoglu et al. 2010). In primary human ovarian granulosa cells, chemerin decreases IGF‐1‐induced thymidine incorporation by decreasing the phosphorylation of ERK signaling pathways (Reverchon et al. 2012). The contradictory results could be due to the activation of different signaling pathways in different cell types.

**Figure 9:**
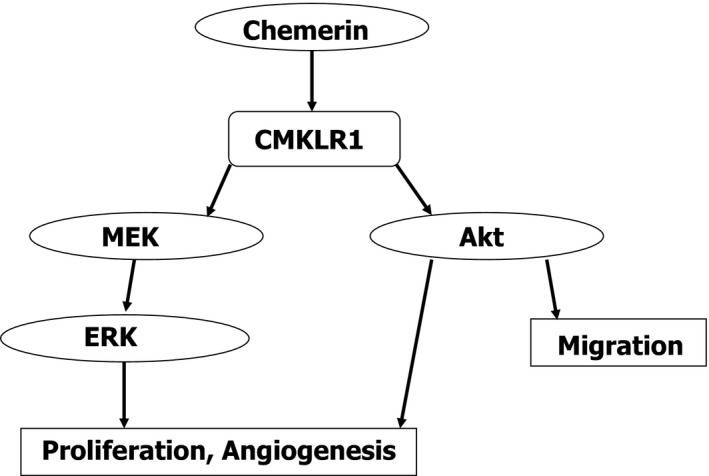
Proposed scheme for chemerin‐stimulated signaling of proliferation and migration. Chemerin promotes angiogenesis and proliferation activity of HUVECs via MEK/ERK and PI3‐K/Akt, whereas chemerin promotes the migration activity only via PI3‐K/Akt.

In this study, the chemerin‐mediated migratory activity of HUVECs was markedly suppressed by the administration of the PI3K inhibitor LY294002 but not the MEK inhibitor PD98059. Whereas, the chemerin‐mediated angiogenic activity and proliferation of HUVECs were markedly suppressed by the administration of either LY294002 or PD98059. These results suggest that PI3‐K/Akt axis is important for endothelial cell invasion and migration in the initial step of angiogenesis, PI3‐K/Akt axis and MEK/ERK axis are important for angiogenesis which depends on endothelial cell viability (Figure 9).

For the first time, we demonstrate that recombinant mature chemerin of 137 aa induces angiogenesis in vivo; however, a limitation of this study is that we used the active form of chemerin at a concentration that is higher than physiological condition. Prochemerin is present at relatively high concentrations in human plasma (6–12 nmol/L, corresponding to 100–200 ng/mL in healthy individuals), but the concentration of bioactive chemerin is negligible under physiological conditions in humans (Ernst and Sinal 2010; Rourke et al. 2013).

There are several limitations in this study. Clinical efficacy of intravitreal anti‐VEGF drugs has been widely demonstrated in several angiogenesis‐driven eye diseases including diabetic retinopathy (Fogli et al. 2018). In this study, we could not conclude whether endogenous chemerin is required for or physiologically important as a driver of angiogenesis. We could not analyze the role of Akt and ERK via CMKLR1 in angiogenesis in vivo. Further studies are required for this problem.

In summary, we have reported here that chemerin promotes the migration of ECs mainly through PI3K/Akt and angiogenesis through Akt and ERK. We have also reported firstly that chemerin can induce angiogenesis via CMKLR1 in human endothelial cells (ECs) in vitro. This study investigates the molecular mechanisms of chemerin in promoting the angiogenesis of ECs via CMKLR1, which plays an important role in neovascularization and reendothelialization. This study may provide a better understanding of the biological functions of chemerin and its potential clinical applications.

## Conflict of Interest

The authors declare that they have no conflicts of interest with the contents of this article.
